# Factors Influencing the Level of Depression and Anxiety of Community-Dwelling Patients with Schizophrenia in China during the COVID-19 Pandemic

**DOI:** 10.3390/ijerph20054376

**Published:** 2023-02-28

**Authors:** Shanshan Chen, Xiaohua Sun, Qisha Zhu, Yuan Zhao, Jinsong Tang, Haidong Song

**Affiliations:** 1 Department of Psychiatry, Sir Run Run Shaw Hospital, School of Medicine, Zhejiang University, Hangzhou 310000, China; 2 Affiliated Mental Health Center, School of Medicine, Zhejiang University, Hangzhou Seventh People′s Hospital, The 4th Clinical College of Zhejiang Chinese Medical University, Hangzhou 310000, China

**Keywords:** schizophrenia, COVID-19, pandemic, anxiety, depression

## Abstract

The coronavirus disease 2019 (COVID-19) poses a huge challenge to global public health. People with schizophrenia living in communities urgently need effective interventions to help them adjust to life and work, but they have not received enough attention. This study aims to assess the prevalence of anxiety and depression symptoms in community-dwelling patients with schizophrenia in China during the epidemic and to explore the possible influencing factors. Methods: Using a cross-sectional survey, we collected 15,165 questionnaires. Assessments included demographic information, concern about COVID-19-related information, sleep status, anxiety and depressive symptoms, and accompanying illnesses. The 7-item Generalized Anxiety Disorder (GAD-7) and the 9-item Patient Health Questionnaire (PHQ-9) were used to evaluate depression and anxiety levels. Group comparison was conducted by *t*-test, ANOVA, or chi-square test wherever suitable, with Bonferroni pairwise correction. Multivariate logistic regression was performed to identify predictors for anxiety and depression. Results: 16.9% of patients had at least moderate anxiety, and 34.9% had at least moderate depression. *T*-test showed that females scored higher on GAD-7 and PHQ-9 than males, and patients without accompanying long-standing diseases, who were not concerned about COVID-19, had lower GAD-7 and PHQ-9 scores. ANOVA showed that participants aged from 30 to 39, with higher education scored higher on GAD-7, and patients with better sleep, and having less concern about COVID-19, had lower GAD-7 and PHQ-9 scores. Regression analysis indicated that participant ages of 30–39 and 40–49 positively predicted anxiety, whereas patient ages of 30–39 years positively predicted depression. Patients with poor sleep, accompanying diseases, and concern about the COVID-19 pandemic were more likely to experience anxiety and depression. Conclusion: During the pandemic, Chinese community-dwelling patients with schizophrenia had high rates of anxiety and depression. These patients deserve clinical attention and psychological intervention, especially those with risk factors.

## 1. Introduction

The discovery and rapid spread of the coronavirus disease 2019 (COVID-19) brought huge challenges to the public health and medical communities around the world [1]. The health effects of this virus are worrisome, including death, a strained healthcare system and economic uncertainty. Likewise, the epidemic may have devastating psychological and social effects [2]. Numerous studies assessed the mental health of the general population during the COVID-19 pandemic [3,4], but there was insufficient research on the emotional impact of schizophrenia patients during the epidemic.

It was reported that patients with schizophrenia had a significantly increased risk of contracting COVID-19 compared to the normal population [5]. High-risk factors included failure to properly recognize self-protection needs and to adhere to preventive behaviors due to impaired cognitive function [6], difficulties in evaluating health information, limitations in access to healthcare [7], and being easily influenced by the ongoing media coverage of the epidemic [8]. Compared with inpatients, community-dwelling patients with schizophrenia lack full-time management of medical care, so community and family support need to play a better role [9]. Therefore, it is necessary to assess the mental health burden of patients with schizophrenia during the COVID-19 pandemic and provide timely community and family support.

Several studies conducted in China in 2020 showed that hospitalized schizophrenic patients with suspected COVID-19 in isolation wards experienced sleep disturbances, significantly higher scores on depression and anxiety scales, and increased stress compared to general hospitalized schizophrenic patients [10,11]. In addition, hospitalized patients with schizophrenia with suspected COVID-19 were reassessed as having significantly increased anxiety symptoms after 10–14 days of isolation [11].

Although community-dwelling patients with schizophrenia did not have as narrow and limited social network connections as long-term hospitalized patients [12], they were relatively underreported during the epidemic. A study shows that many community schizophrenia patients, despite being stable, still have some psychiatric symptoms that affect their lives to some extent [12,13]. An outpatient health study showed that only about 25% of patients with schizophrenia achieved functional remission during the 3-year follow-up period [14]. Community-dwelling patients with schizophrenia are in urgent need of effective intervention methods to help them adjust to life and work during a pandemic. Therefore, we intended to explore the psychological burden of community-dwelling patients with schizophrenia during the pandemic. One study showed that community-dwelling patients with schizophrenia or bipolar disorder experienced more severe anxiety and depressive symptoms during the urban lockdown compared to community healthy controls [15]. However, the report did not analyze anxiety and depressive symptoms separately in patients with schizophrenia during the outbreak. A Spanish study showed that compared to the control group, community-dwelling patients with schizophrenia (*n* = 42) experienced significantly higher scores in Hospital Anxiety and Depression Scale Anxiety (HADS-A) and Hospital Anxiety and Depression Scale Depression (HADS-D) during the COVID-19 pandemic [16]. Moreover, 40.8% of community-dwelling patients with schizophrenia (*n* = 76) reported depression and 32.9% reported anxiety [17]. Overall, current research on depression and anxiety in community-dwelling schizophrenics during the epidemic period is insufficient, and inadequate sample sizes are a shortcoming.

Given the evolving and unpredictable duration of COVID-19, using a cross-sectional, web-based and large sample study, the first objective of this study was to examine the mental health burden of community-dwelling patients with schizophrenia during the COVID-19 outbreak, and the second objective was to analyze potential influencing factors. This study assessed the impact of the COVID-19 pandemic crisis on the mental health of community-dwelling patients with schizophrenia. A study of community-dwelling patients with schizophrenia would help provide effective psychological screening and interventions. We hypothesized that community-dwelling patients with schizophrenia during the COVID-19 pandemic had varying degrees of anxiety and depressive symptoms, and that middle age, poor sleep, concerns about epidemic information, and concomitant other long-standing illnesses would have an impact on depression and anxiety.

**Figure 1:**
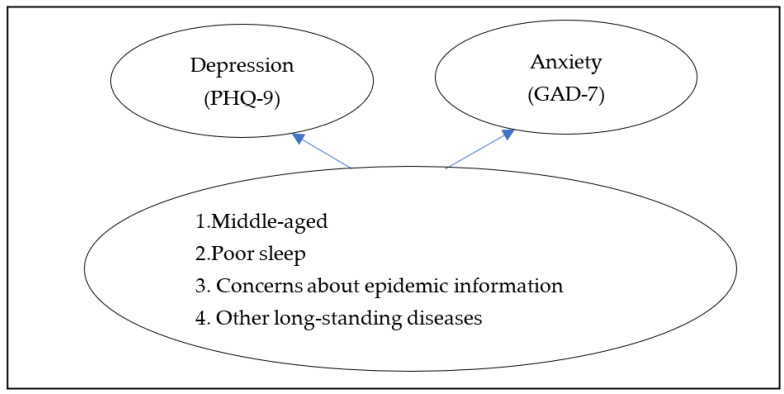
Theoretical Framework.

This article is divided into five parts. The first part introduces the background of the study and the research hypothesis, the second part describes the details of the research methodology. The third part contains the results of this study and the corresponding explanations. The fourth part discusses the results of this study. The last part contains the conclusion, shortcomings and directions for future research. Figure 1 shows the theoretical framework.

## 2. Literature Review

### 2.1. Sleep Status

In our study, sleep status refers to the sleep level of the subjects during the past 12 months. If the sleep status was good most of the year, a better status is recorded, and if the sleep status was bad most of the year, the status “poor” is chosen. Poor sleep includes sleep problems such as difficulty falling asleep or staying asleep. Schizophrenia is commonly accompanied by sleep disturbances [18]. There is an important relationship between sleep disorder and depression [19]. People with persistent insomnia have higher levels of severe depression, general anxiety, and panic [20]. A small foreign sample study showed that community patients with schizophrenia or bipolar disorder reported more anxiety and depression and experienced more sleep disturbances compared to normal individuals during the COVID-19 epidemic and embargo, but the study did not analyze the underlying factors influencing depressed and anxious mood and the grouping did not distinguish patients with schizophrenia from those with bipolar disorder [15].

### 2.2. Accompanying Long-Standing Diseases

A long-standing disease is a group of non-communicable diseases that have a long history of onset and cannot be cured once they develop. The common ones include coronary heart disease, stroke, hypertension, malignant neoplasm, diabetes, chronic respiratory diseases, etc. The risk of schizophrenia patients suffering from diabetes [21], and hypertension [22] is higher than that of the general population. There is a complex association between depression and chronic diseases [23]; for example, diabetes increases the risk of depressive symptoms [24], and depressive symptoms are associated with hypertension [25]. However, there are no studies related to mood and chronic illness in large samples of community schizophrenics, and certainly even fewer investigations during the COVID-19 epidemic.

### 2.3. Concern about COVID-19 and the Degree of Concern about the COVID-19 Epidemic

We explained the level of concern about the epidemic when we collected the questionnaire. Less attention refers to the occasional check on outbreak-related information. General attention refers to a moderate level of attention. More attention means that you are often proactive in getting information and keeping tabs on the progress of the outbreak. There is a lot of information about the epidemic, including inaccurate information. When people pay more attention to negative information, there will be more adverse emotional reactions [26]. However, similar studies conducted on patients with schizophrenia in the community are not currently found.

## 3. Methods

### 3.1. Research Approach

In this study, the research method we used was a questionnaire survey. This is a low-cost method, commonly used for a wide range of surveys [27,28].

### 3.2. Questionnaire Development

This study consists of 2 parts. The first part contains demographic data and some other information, including sleep status, and other accompanying long-standing diseases, and concerns about COVID-19. We defined “ long-term diseases “ and explained “ sleep status” and “concerns about COVID-19” above, in the literature review. The options all have 2–3 simple categories. The second part of the questionnaire, which is the main part of our study, contains the Depression Scale and the Anxiety Scale, which are widely used scales for measuring depression and anxiety worldwide [29]. The research questionnaire is in Supplementary Materials File S1. In addition, a pilot test was conducted to ensure that participants understood the purpose and content of the questionnaire before it was widely distributed. In the pilot test, the questionnaire was reviewed by three subject matter experts and this provided adoptable recommendations [30].

### 3.3. Measurement

#### 3.3.1. Anxiety Scale

The 7-item Generalized Anxiety Disorder (GAD-7) was adopted to assess the severity of self-reported anxiety [31]. It is composed of 7 items to evaluate how often over the past two weeks the patient has suffered from various issues, such as “difficulty in relaxing” or “excessive worry”. Response categories are “not at all”, “several days”, “more than one week”, and “nearly every day”, scored as 0, 1, 2, and 3, respectively. The total score of the GAD-7 is calculated by summing each item score. The total score ranges from 0 to 21, with a score of 5 indicating that the patient has anxiety. A score of 5, 10 or 15 represents the threshold for “mild”, “moderate” or “severe” anxiety, respectively. Studies showed that the scale has good internal consistency (Cronbach’s α = 0.92), and the test-retest reliability coefficient was 0.83. When the decomposition value was 10 points, the sensitivity was 89% and the specificity was 82% [32]. In this study, we conducted a reliability analysis yielding Cronbach’s α = 0.893. In the validity analysis, KMO = 0.915 and Bartlett’s Test of Sphericity <0.001. The cumulative sum of squares of the total variance of GAD-7 item 1 to item 7 was 61.940%, 71.599%, 78.494%, 84.781%, 90.333%, 95.505%, and 100.000%, respectively. The standard loadings for items 1 to item 7 of the GAD-7 were 0.706, 0.805, 0.800, 0.827, 0.806, 0.783, and 0.775, respectively, all of which were above 0.7.

#### 3.3.2. Depression Scale

The 9-item Patient Health Questionnaire (PHQ-9), also with good reliability and validity, was employed to screen depressive disorder and measure the severity of symptoms [33]. PHQ-9 is made up of 9 items to evaluate how often over the past two weeks the patient has suffered from nine issues, including depressed mood and anhedonia. Response categories are “not at all”, “several days”, “more than one week”, and “nearly every day”, scored as 0, 1, 2, and 3, respectively. The total score ranges from 0 to 27, with a score of 5 indicating that the patient has depression. A score of 5, 10, 15 or 20 represents the threshold for “mild”, “moderate”, “severe” or “extremely severe” depression, respectively. Nine items of PHQ-9 include anhedonia, depressed mood, sleep disturbance, fatigue, appetite changes, low self-esteem, concentration problems, psychomotor disturbances, and suicidal ideation. In this study, we conducted a reliability analysis yielding Cronbach’s α = 0.897. In the validity analysis, KMO = 0.926 and Bartlett’s Test of Sphericity <0.001. The cumulative sum of squares of the total variance for PHQ-9 item 1 to item 9 was 56.065%, 66.292%, 73.043%, 78.714%, 83.856%, 88.506%, 92.671%, 96.552, and 100.000%, respectively. The standard loadings for items 1 to 9 of PHQ-9 were 0.789, 0.799, 0.718, 0.788, 0.753, 0.776, 0.764, 0.754, and 0.571, respectively, and almost all were above 0.7 except the last item.

### 3.4. Sampling and Data Collection

This cross-sectional questionnaire was conducted in a prefecture-level city in Hangzhou, China from April 7 to May 10, 2020. According to the local area division, the city has 13 districts, counties (cities) and 2 functional areas. The researchers attempted to contact all schizophrenia patients registered with the local health system and eventually obtained 15,165 questionnaires from schizophrenia patients who were eligible for enrollment, of whom 2047 subjects were not successfully enrolled because of their unstable condition.

We designed the content of the questionnaire. Patients with schizophrenia living in the communities under their respective management were contacted by physicians engaged in the prevention and treatment of mental illness in each district, county, and prefecture through telephone, home visits, and outpatient clinics, and the questionnaires were completed online by the physicians after obtaining patient information. Prior to the start of the study, local physicians were trained with detailed information on the questionnaire items and quality control of data collection. During the survey process, physicians were responsible for explaining the purpose of the study, presenting the questionnaire content, and ensuring that participants fully understood it.

Inclusion criteria were: living in China, being able to communicate normally and meeting the Diagnostic and Statistical Manual of Mental Disorders IV (DSM-IV) diagnosis of schizophrenia, and keeping the disease stable over the course of a year. Exclusion criteria were: in the acute phase of schizophrenia, accompanying serious medical illness. The 15,165 subjects included 7010 male participants (46.2%) and 8155 female participants (53.8%). Age ranged from 10 to 96 years old, and the mean age was 55.4 years ± 13.9 S.D.

### 3.5. Ethical Approval

The study complied with the ethical standards of the Declaration of Helsinki and was approved by the Ethics Committee of Hangzhou Seventh People’s Hospital (No. 2020035). All participants provided written informed consent. Subjects who communicated face-to-face signed a paper informed consent form, and those who communicated by phone signed an electronic informed consent form through an online platform. Because the participants in this study included minors, informed consent was obtained from their parents and/or legal guardians.

### 3.6. Statistical Analysis

Data analyses were conducted using IBM SPSS Statistics software version 19.0 (IBM Corporation, Armonk, NY, USA). Categorical data were described by the case numbers (percentage) and quantitative data as mean score ± S.D. Group comparison was conducted by *t*-test, ANOVA, or chi-square test wherever suitable, and pairwise comparison was conducted by Bonferroni test. In order to look for the score differences of PHQ-9 and GAD-7, patients with schizophrenia were grouped into six sub-groups by demographic and clinical status, namely sex, age, marital status, educational level, sleep status, and other accompanying long-standing diseases. Multivariate logistic regression models were performed to identify independent predictors for anxiety and depression, respectively. GAD-7 and PHQ-9 scores were first transformed into binary variables with the threshold of moderate anxiety or depression and then served as dependent variables in the respective regression models. Anxiety was divided into mild anxiety and moderate and above anxiety; moderate and above anxiety was defined as a patient with a score of >9. Depression was divided into mild depression and moderate and above depression; moderate and above depression was defined as a patient with a score of >9. Sex, age, marital status, educational level, sleep status, other accompanying long-standing diseases, concern about COVID-19, and the degree of concern about COVID-19 served as independent variables. Statistical significance was set at a two-sided *p*-value < 0.05.

## 4. Results

The completed questionnaire data were entered into an SPSS file. Since the management of schizophrenia in the community is systematically standardized, with specific community physicians managing regular patients over time, and maintaining follow-up visits and follow-up, subjects who agreed to be enrolled provided valid and complete data. There were no missing values and no outliers were found in this study. We performed validation factor analysis to test the reliability and validity of the depression scale and anxiety scale in this study. This study wanted to explore the potential influences that affect patients with schizophrenia presenting moderate and higher levels of depression and anxiety, consistent with exploring the relationship between dichotomous dependent variables and independent variables, so a dichotomous logistic regression model was used for analysis. Previous studies have shown that roughly 80% of patients with schizophrenia have reported depression [34]. Although there was an increase in the proportion in the present findings, patients have been receiving regular attention to mood changes from community healthcare providers during routine management of the illness, and mild depression has less impact compared to moderate and higher depression. However, some studies have shown that patients with moderate depression are at significantly increased risk for suicide [35] and that major depressive disorder is a significant factor in suicide in patients with schizophrenia [36]. In addition, some studies have shown that the worsening of anxiety symptoms is a strong predictor of medication switching during treatment, suggesting that severe anxiety enhances the adverse effects of treatment management [37]. Therefore, we prefer to explore the potential influences affecting moderate and higher levels of anxiety and depression, which certainly does not make us neglect to focus on patients with mild depression and anxiety.

### 4.1. General Distribution of Patient’s Anxiety or Depression

Among 15,165 cases of patients with schizophrenia, the mean score of GAD-7 and PHQ-9 were 8.1 ± 2.1 and 10.4 ± 2.7, respectively, and the prevalence of anxiety and depression was 100%. The proportion with mild anxiety was 83.2%, moderate anxiety accounted for 15.7%, and 1.2% had severe anxiety. Meanwhile, 65.1% of participants had minimal symptoms of depression, the moderate depression rate was 25.7%, the proportion with severe depression was 8.2%, and 1.0% had extremely serious depression. See Table 1 for details.

### 4.2. Subgroup Analyses of Questionnaire Scores

Regarding the GAD-7 score, group comparison showed that female patients were more anxious than men (t = −2.03, *p* = 0.042). Patients in the 30–39 age group were more anxious than other age groups (F = 2.84, *p* = 0.014). Patients with university degrees or above had the highest anxiety (F = 4.08, *p* = 0.007). Patients with other accompanying long-standing diseases were more anxious than those without other diseases (t = 4.18, *p* < 0.001). Patients with poor sleep (F = 158.87, *p* < 0.001) had high levels of anxiety. Similarly, PHQ-9 results showed that female patients were more depressed than men (t = −3.27, *p* = 0.001). Patients with poor sleep (F = 284.00, *p* < 0.001) had higher levels of depression. Patients with other accompanying long-standing diseases were more depressed than those without other diseases (t = 6.86, *p* <0.001). See Table 2 for details.

### 4.3. Concern about the COVID-19 Pandemic

*T*-tests showed that patients who were concerned about COVID-19 scored higher on GAD-7 (t = 8.17, *p* < 0.001) and PHQ-9 (t = 2.29, *p* = 0.022), respectively, compared to those who were not concerned about COVID-19. ANOVA showed those with general concern about COVID-19 scored higher on GAD-7 (F = 93.19, *p* < 0.001) and PHQ-9 (F = 95.30, *p* < 0.001), respectively, than those with less or more concern about COVID-19. Detailed data is presented in Table 3.

### 4.4. Multivariate Logistic Regression Models for Anxiety and Depression among Community-Dwelling Patients with Schizophrenia

The details about the multivariate analyses of predictors with logistic regression models for anxiety and depression are shown in Table 4. Our study showed that people in the 30–39 (OR:1.14; 95% CI [0.39, 3.4]; *p* = 0.811) and 40–49 (OR:1.16; 95% CI [1.03, 1.32]; *p* = 0.018) age groups, with other accompanying long-standing diseases (OR:1.15; 95% CI [1.03, 1.29]; *p* = 0.013), who were concerned about the COVID-19 pandemic (OR:1.44; 95% CI [1.26, 1.65]; *p* < 0.001), were more likely to experience anxiety. Good sleep (OR:0.23; 95% CI [0.19, 0.28]; *p* < 0.001) can reduce the risk of anxiety. Meanwhile, patients aged 30–39 (OR:1.23; 95% CI [1.09, 1.38]; *p* = 0.001), with other accompanying long-standing diseases (OR:1.29; 95% CI [1.18, 1.42]; *p* < 0.001), who were concerned about the COVID-19 pandemic (OR:1.49; 95% CI [1.34, 1.64]; *p* < 0.001) were more likely to experience depression. Good sleep (OR:0.26; 95% CI [0.22, 0.31]; *p* < 0.001) can reduce the risk of depression. Other independent variables are non-significant predictors in the logistic regression model for anxiety and depression (Table 4). Interestingly, we performed regression analysis separately for males and females and found that male patients with a partner (OR:0.88; 95% CI [0.79, 0.97]; *p* = 0.013) had a lower risk of depression (Table 5), and the remaining results did not change much. Detailed data is shown in Table 4 and Table 5.

## 5. Discussion

In the current study, all community-dwelling patients with schizophrenia had different degrees of anxiety and depression during the epidemic period. Moderate to severe anxiety accounted for 16.9%, and the percentage of moderate and above depression was 34.9%, which partly meets our first hypothesis. Regression analysis showed that ages from 30 to 39, poor sleep, other accompanying long-standing diseases, and concern about the COVID-19 pandemic are potential factors of depression and anxiety, which is consistent with our second hypothesis.

15,165 cases of community-dwelling patients with schizophrenia in this survey all had mild to severe anxiety and depression, which is supported by previous research. Compared to the control group, community-dwelling patients with schizophrenia experienced significantly higher scores in HADS-A and HADS-D during the COVID-19 pandemic [16]. Moreover, 40.8% of community-dwelling patients with schizophrenia (*n* = 76) reported depression and 32.9% reported anxiety [17].

In this study, the GAD-7 and PHQ-9 scores of schizophrenia patients in the 30–39 age group were higher than those of other age groups. Compared with patients with schizophrenia who were older than 60 years, patients aged from 30 to 39 and from 40 to 49 were more likely to experience anxiety, and compared with patients older than 60 years old, patients aged 30–39 scored higher in depression. Previous studies showed that during the COVID-19 pandemic, people aged 30–49 had higher scores on epidemic knowledge and paid more attention to epidemic information, which may increase the risk of depression and anxiety [38]. As we know, the unemployment rate of schizophrenic patients is high, ranging from 80% to 90%, resulting in limited economic income [39]. Middle-aged patients undergo a period of shouldering societal and familial responsibility, although their physiological function is gradually declining. During the epidemic, due to limited social activities [40], the financial resources of patients may be greatly affected, which may lead to greater stress among community schizophrenic patients, resulting in more obvious symptoms of anxiety and depression.

We found that patients with a partner had less serious depression symptoms than those without any partner. Studies have shown that schizophrenia is usually associated with severe damage in many areas of life, including intimacy and social adjustment [41]. Patients with schizophrenia, especially men, are less likely to get married than others [42,43]. Several studies showed that being unmarried is a sociodemographic risk factor for schizophrenia [42,44], and for patients with schizophrenia, being single itself may present a risk of adverse outcomes [45]. Previous studies showed that married patients with schizophrenia or schizoaffective disorder evaluate their quality of life higher than single subjects, and have fewer suicidal ideations than divorced, widowed, or separated subjects [46]. When the COVID-19 pandemic occurred, the situation changed. The epidemic posed a serious threat to patients’ children and families. The consequences of these difficulties may be long-term, partly because environmental risks penetrate the structure and process of the family system [47], while the patients’ partners can take more risks together, so as to reduce the pressure of the patient. Moreover, male schizophrenia patients who were currently in marital status had the least disease-related symptoms [48].

The results of this study revealed that schizophrenic patients with poor sleep had more severe anxiety and depression symptoms, which was similar to previous studies [49]. Approximately 90% of people diagnosed with depression [50] and approximately 70% of patients experiencing anxiety [51] self-reported lack of sleep. Substantial evidence suggests that sleep disturbance is a prodromal symptom of recurrent depressive episodes [52,53]. In addition, depression, anxiety, fear, etc., are more likely to cause sleep problems [54].

We found patients with schizophrenia accompanied by other long-standing diseases were at higher risk of anxiety and depression. A Turkish study showed that the general population with chronic diseases will be more seriously affected by depression and anxiety symptoms, and that other accompanying chronic diseases was a risk factor for anxiety. It may be that patients with schizophrenia are more sensitive and aware of how their body feels [55].

The GAD-7 and PHQ-9 scores of community-dwelling patients with schizophrenia who were concerned about COVID-19 were higher than those who did not express concern. Besides, the group comparison above showed that the anxiety and depression symptoms of patients with general concern about the epidemic were more serious than those with less or more concern. For the first result, this may be because patients who did not express concern about COVID-19 at all could not understand the severity of the epidemic, and were less worried about the health problems of the epidemic, which led to insignificant anxiety and depression symptoms. For those who were occasionally concerned about the epidemic, aware of the methods of controlling infectious diseases including risk communication, hygiene habits, social distancing, and believing that vaccines were safe and effective [56], their anxiety and depression were naturally reduced. Patients who were moderately concerned but not thoroughly concerned about the outbreak experienced the most severe symptoms of depression and anxiety, probably because they were not ignorant, but overwhelmed by seeing more information on COVID-19 [40]. Some of the information or relevant knowledge they obtained may have been superficial, especially in uncertain periods. Conspiracy theories and rumors were particularly popular during the pandemic. For example, some people saw many rumors but did not further obtain rumor refutation information. Social media may be double-sided as an information source under the influence of some people [56]. Since the information obtained by patients was too much but not in-depth enough and true information could not be distinguished from false, patients were more likely to feel a lack of control, producing more serious anxiety and depression.

## 6. Conclusions, Limitations, and Future Research

### 6.1. Conclusions

This was the first study to investigate the psychological burden of community-dwelling patients with schizophrenia with a large sample during the COVID-19 epidemic. The study showed that anxiety and depression symptoms of different degrees generally appeared in community schizophrenia during the epidemic period, which confirmed that the epidemic had a certain impact on the mental health of patients. In addition, we found that in the current study, the 30–39 age group, sleeping poorly, having other accompanying long-standing diseases, and concern about COVID-19 were risk factors for anxiety or depression. Besides, patients with general concerns about COVID-19 had more severe symptoms of depression and anxiety. Living with a partner was a protective factor for depression in male patients.

As our findings showed, patients with schizophrenia had obvious psychological stress responses such as anxiety and depression during the epidemic. Previous research has shown that pandemics can lead to unemployment and family poverty, separation of family members, and social isolation [56]. At the same time, the number of patients being screened for safety reasons decreases, and individuals with psychiatric symptoms may have difficulty accessing medical assistance [55]. Therefore, we need to focus on the mental health of patients in a timely and adequate manner. These patients are at risk of experiencing more severe consequences after stimulation, such as worsening of psychiatric symptoms and disease relapse [57]. Community grassroots medical staff and members of care and rescue groups need to focus on follow-up and emotional counseling for patients with the above characteristics. For example, patients with the above characteristics should be surveyed with a higher frequency of questionnaires to understand their emotional state in time. In serious cases, medication and psychological intervention should be carried out. In addition, community workers need to guide patients to correctly understand COVID-19 and reduce unnecessary concerns. Moreover, primary medical staff need to pay more attention to the sleep status of schizophrenia patients in the community, including drug adjustment and behavioral intervention. Finally, it is important to actively treat other concomitant diseases of patients with schizophrenia. Of course, this mental health program should not increase the burden on healthcare providers or the risk of spreading the infection to others [56].

### 6.2. Limitations and Future Research

This survey also has certain limitations. First, due to the special period of epidemic prevention and control, the patient’s mental health questionnaire survey was delivered by home visits, telephone calls, outpatient clinics, etc. Different delivery forms may cause misunderstandings and inaccuracy of the results. Although physicians had unified the standards for questionnaire interviews, different transmission forms may still cause misunderstandings and inaccurate results. In this study, telephone or face-to-face interviews were used. Face-to-face communication may help patients understand better than phone conversations, and the results obtained would be relatively more accurate. Second, in order to obtain patient information conveniently, all results were derived from self-report scales; although physicians tried their best to explain the items, this could still lead to biases in patients’ recall. Third, this study was mainly based on the evaluation of patients by local physicians practicing mental illness prevention and the results may be affected by their subjective evaluation. Finally, we did not evaluate the depression or anxiety symptoms of these patients before the outbreak, which leads to a lack of longitudinal comparison. In the future, we hope to carry out longitudinal research and a more detailed assessment of the emotion and cognition of patients with schizophrenia in the community to help improve their community and family support.

## Figures and Tables

**Table 1:** The severity of depression and anxiety in patients during COVID-19.

Severity	GAD-7		Severity	PHQ-9	
	*n*	(%)		*n*	(%)
Mild	12,612	83.2	Mild	9867	65.1
Moderate	2377	15.7	Moderate	3902	25.7
Severe	176	1.2	Severe	1249	8.2
			Extremely serious	147	1.0
Total	15,165	100		15,165	100

*n*: number; SD, standard deviation; GAD-7: the 7-item Generalized Anxiety Disorder; PHQ-9: the 9-item Patient Health Questionnaire; COVID-19: 2019 Coronavirus Disease.

**Table 2:** Comparison of GAD-7 and PHQ-9 scores in patients with different characteristics.

Characteristic	*n* (%)	GAD-7 (Mean ± SD)	*F*/*t*/*χ*^2^	*p*	PHQ-9 (Mean ± SD)	*F*/*t*/*χ*^2^	*p*
Sex			−2.03	0.042		−3.27	0.001
Male (%)	7010 (46.2)	8.1 ± 2.1			10.3 ± 2.5		
Female (%)	8155 (53.8)	8.2 ± 2.2			10.5 ± 2.8		
Age (years)			2.84	0.014		1.79	0.111
≤18	24 (0.2)	8.1 ± 1.9			10.2 ± 2.3		
19~29	508 (3.3)	8.0 ± 2.2			10.3 ± 2.9		
30~39	1647 (10.9)	8.3 ± 2.3			10.6 ± 3.0		
40~49	2642 (17.4)	8.2 ± 2.1			10.4 ± 2.6		
50~59	4433 (29.2)	8.1 ± 2.1			10.4 ± 2.6		
≥60	5911 (39.0)	8.1 ± 2.1			10.4 ± 2.7		
Marital status			1.69	0.092		0.90	0.368
With partner	8607 (56.8)	8.2 ± 2.2			10.4 ± 2.7		
Without partner	6558 (43.2)	8.1 ± 2.1			10.4 ± 2.6		
Education (years)			4.08	0.007		0.38	0.769
Illiteracy	2528 (16.7)	8.1 ± 2.2			10.4 ± 2.8		
Primary school	4786 (31.6)	8.1 ± 2.1			10.4 ± 2.7		
Junior high school	7069 (46.6)	8.2 ± 2.1			10.4 ± 2.6		
University and above	782 (5.2)	8.3 ± 2.2			10.4 ± 2.5		
Sleep status			158.87	<0.001		284.00	<0.001
Better	4256 (28.1)	7.8 ± 1.6			9.8 ± 1.8		
Normal	10,243 (67.5)	8.2 ± 2.2			10.6 ± 2.7		
Poor	666 (4.4)	9.1 ± 3.2			12.0 ± 4.4		
Accompanying other long-standing disease			4.18	<0.001		6.86	<0.001
Yes	2648 (17.5)	8.3 ± 2.3			10.7 ± 3.0		
No	12,517 (82.5)	8.1 ± 2.1			10.3 ± 2.6		

*n*: number; SD, standard deviation; GAD-7: the 7-item Generalized Anxiety Disorder; PHQ-9: the 9-item Patient Health Questionnaire; COVID-19: 2019 Coronavirus Disease.

**Table 3:** Patient’s concern about the COVID-19 pandemic.

	*n* (%)	GAD-7 (Mean ± SD)	*F*/*t*	*p*	PHQ-9 (Mean ± SD)	*F*/*t*	*p*
Concern about COVID-19			8.17	<0.001		2.29	0.022
Yes	12,722 (83.9)	8.2 ± 2.1			10.4 ± 2.6		
No	2443 (16.1)	7.8 ± 2.0			10.3 ± 2.9		
Total	15,165 (100)						
The degree of concern about COVID-19			93.19	<0.001		95.30	<0.001
Less	12,055 (79.5)	8.0 ± 2.0			10.2 ± 2.5		
General	2803 (18.5)	8.6 ± 2.5			11.0 ± 3.1		
More	307 (2.0)	8.4 ± 2.8			10.5 ± 3.2		
Total	15,615 (100)						

*n*: number; SD, standard deviation; GAD-7: the 7-item Generalized Anxiety Disorder; PHQ-9: the 9-item Patient Health Questionnaire; COVID-19: 2019 Coronavirus Disease.

**Table 4:** Logistic regression analysis of anxiety and depression in patients with schizophrenia in the community.

		Anxiety ^a^		Depression ^b^	
		OR(95% CI)	*p*	OR(95% CI)	*p*
Age	≤18	1.14 (0.39–3.4)	0.811	0.86 (0.35–2.09)	0.734
	19~29	0.94 (0.72–1.21)	0.620	0.92 (0.75–1.12)	0.387
	30~39	1.26 (1.09–1.46)	0.002	1.23 (1.09–1.38)	0.001
	40~49	1.16 (1.03–1.32)	0.018	1.04 (0.94–1.15)	0.488
	50~59	1.03 (0.92–1.15)	0.614	1.01 (0.93–1.10)	0.786
	≥60	1.00		1.00	
Sleep status	Better	0.23 (0.19–0.28)	<0.001	0.26 (0.22–0.31)	<0.001
	Normal	0.47 (0.40–0.56)	<0.001	0.54 (0.46–0.63)	<0.001
	Poor	1.00		1.00	
Accompanying other long-standing diseases	Yes	1.15 (1.03–1.29)	0.013	1.29 (1.18–1.42)	<0.001
	No	1.00		1.00	
Concerning about COVID-19	Yes	1.44 (1.26–1.65)	<0.001	1.49 (1.34–1.64)	<0.001
	No	1.00		1.00	
The degree of concern about COVID-19	Less	0.78 (0.57–1.05)	0.095	0.85 (0.67–1.08)	0.188
	General	1.38 (1.01–1.87)	0.041	1.54 (1.20–1.99)	0.001
	More	1.00		1.00	

OR: odds ratio; COVID-19: 2019 Coronavirus Disease. ^a^ Anxiety was divided into mild anxiety and moderate and above anxiety; moderate and above anxiety was defined as a patient with a score of >9. ^b^ Depression was divided into mild depression and moderate and above depression; moderate and above depression was defined as a patient with a score of >9.

**Table 5:** Logistic regression analysis of anxiety and depression in male and female patients with schizophrenia in the community.

		Anxiety ^a^		Depression ^b^	
		OR(95% CI)	*p*	OR(95% CI)	*p*
Male (*n* = 7010)					
Marital status	With partner	/	/	0.88 (0.79–0.97)	0.013
	Without partner	/	/	1.00	
Sleep status	Better	0.30 (0.22–0.41)	<0.001	0.31 (0.23–0.40)	<0.001
	Normal	0.54 (0.41–0.72)	<0.001	0.61 (0.47–0.79)	<0.001
	Poor	1.00		1.00	
Accompanying other long-standing diseases	Yes	/	/	1.37 (1.20–1.57)	<0.001
	No	/	/	1.00	
Concerning about COVID-19	Yes	1.36 (1.12–1.65)	0.002	1.48 (1.28–1.72)	<0.001
	No	1.00		1.00	
The degree of concern about COVID-19	Less	0.66 (0.44–1.01)	0.055	0.69 (0.49–0.97)	0.032
	General	1.18 (0.77–1.82)	0.443	1.33 (0.94–1.90)	0.112
	More	1.00		1.00	
Female (*n* = 8155)					
Sleep status	Better	0.20 (0.16–0.26)	<0.001	0.24 (0.19–0.30)	<0.001
	Normal	0.44 (0.35–0.55)	<0.001	0.50 (0.41–0.61)	<0.001
	Poor	1.00		1.00	
Accompanying other long-standing diseases	Yes	1.16 (1.00–1.34)	0.046	1.21 (1.07–1.36)	0.002
	No	1.00		1.00	
Concerning about COVID-19	Yes	1.55 (1.29–1.86)	<0.001	1.53 (1.33–1.75)	<0.001
	No	1.00		1.00	
The degree of concern about COVID-19	Less	0.85 (0.55–1.30)	0.454	1.00 (0.71–1.41)	0.997
	General	1.55 (1.00–2.40)	0.050	1.75 (1.23–2.50)	0.002
	More	1.00		1.00	

OR: odds ratio; COVID-19: 2019 Coronavirus Disease. ^a^ Anxiety was divided into mild anxiety and moderate and above anxiety; moderate and above anxiety was defined as a patient with a score of >9. ^b^ Depression was divided into mild depression and moderate and above depression; moderate and above depression was defined as a patient with a score of >9.

## Data Availability

The data presented in this study are available on request from the corresponding author. The data are not publicly available due to the protection of personal data.

## Supplementary Materials

The following supporting information can be downloaded at: https://www.mdpi.com/article/10.3390/ijerph20054376/s1, File S1: Research Questionnaire.
