# Supplementary material for: Factors Influencing the Level of Depression and Anxiety of Community-Dwelling Patients with Schizophrenia in China during the COVID-19 Pandemic
Source: Int J Environ Res Public Health. 2023 Feb 28;20(5):4376. doi: 10.3390/ijerph20054376 (PMC10001627; doi:10.3390/ijerph20054376)
Supplement: Supplementary file 1 [file ijerph-20-04376-s001.zip › File S1.pdf]

Research Questionnaire

Part I Demographic information and general information

1. What is your gender?

A. Male B. Female

2. What is your age?

A. ≤18

B. 19~29

C. 30~39

D. 40~49

E. 50~59

3. What is your marital status?

A. With partner

B. Without partner

4. What is your education level?

A. Illiteracy

B. Primary school

C. Junior high school

D. University and above

5. How about your sleep status during the past 12 months?

A. Better

B. Normal

C. Poor

6. Do you have other accompanying long-standing diseases besides mental illness?

A. Yes

B. No

7. Do you concern about the COVID-19?

A. Yes

B. No

8. How concerned are you about the COVID-19?

A. Less

B. General

C. More

Part II Depression Scale and Anxiety Scale

1) The 7-item Generalized Anxiety Disorder (GAD-7)

There are 7 words below. Please read each carefully to understand the meaning. There are four grades after each word, which means: never; A few days; More than half of the days; Almost every day. According to your actual situation in the last two weeks, tick "√" under the appropriate score of 0-3 points in the score column.

	never	A few days	More than half of the days	Almost every day
1 . Feel uneasy, worried, irritable or irritable …………	0	1	2	3
2 . Unable to stop or control worry ………………………	0	1	2	3
3 . Too worried about all kinds of things ……………………	0	1	2	3
4 . Very nervous, unable to relax ……………………………	0	1	2	3
5 . No appetite or overeating……………………………………	0	1	2	3
6 . I am so anxious that I can't sit still………………………	0	1	2	3
7 . Worried that something bad would happen…………..	0	1	2	3

2) The 9-item Patient Health Questionnaire (PHQ-9)

There are 9 words below. Please read each one carefully to understand the meaning. There are four grades after each word, which means: never; A few days; More than half of the days; Almost every day. According to your actual situation in the last two weeks, mark "√" under the appropriate score of 0-3 points in the score column.

	never	A few days	More than half of the days	Almost every day
1 . Not interested in things ………………………………	0	1	2	3
2 . Feeling depressed, depressed and hopeless …………..	0	1	2	3
3 . Can't sleep or sleep too long…………………………………	0	1	2	3
4 . Feeling tired or lacking energy……………………………	0	1	2	3

5 .	No appetite or overeating……………………………………	0	1	2	3
6 .	Feeling guilty about yourself or feeling like a loser or causing family failure…	0	1	2	3
7 .	Unable to concentrate when doing things, such as reading newspapers or watching TV ……………………	0	1	2	3
8 .	Walking or talking is equivalent to slow or unusual excitement and walking ……………	0	1	2	3
9 .	It's better to die or hurt yourself ………………………………	0	1	2	3
